# Assessing efficacy and safety of dynamic scalp acupuncture for post-stroke rehabilitation: systematic review and meta-analysis protocol

**DOI:** 10.3389/fneur.2026.1750422

**Published:** 2026-07-17

**Authors:** Sang-Joon An, Woo-Chul Shin, Jungbin Song, Jae-Heung Cho, Won-Seok Chung, Mi-Yeon Song, Hyungsuk Kim

**Affiliations:** 1 Department of Clinical Korean Medicine, Graduate School, Kyung Hee University, Seoul, Republic of Korea; 2 Department of Korean Medicine Rehabilitation, Kyung Hee University College of Korean Medicine, Kyung Hee University Korean Medicine Hospital, Seoul, Republic of Korea; 3 Department of Herbal Pharmacology, College of Korean Medicine, Kyung Hee University, Seoul, Republic of Korea

**Keywords:** dynamic scalp acupuncture, meta-analysis, rehabilitation, stroke, systematic review

## Abstract

Stroke is a leading cause of long-term disability worldwide highlighting the need for effective rehabilitation strategies to improve functional recovery. Dynamic Scalp Acupuncture (DSA), which combines scalp acupuncture with concurrent task-oriented training, has emerged as a potentially beneficial intervention for stroke rehabilitation. However, evidence regarding DSA has not been systematically synthesized as a distinct task-integrated rehabilitation approach. This protocol outlines a systematic review and meta-analysis of randomized controlled trials (RCTs) to evaluate the efficacy and safety of DSA in stroke rehabilitation. This study will be conducted in accordance with the Preferred Reporting Items for Systematic Review and Meta-Analysis Protocols (PRISMA-P) guidelines. A comprehensive literature search will be performed in international databases, including PubMed, Embase, the Cochrane Central Register of Controlled Trials, and the Cumulative Index to Nursing and Allied Health Literature; Chinese databases, including the China National Knowledge Infrastructure and Wanfang; and Korean databases, including the Research Information Sharing Service, Korean Studies Information Service System, and Korea Medical database. Eligible studies will be RCTs evaluating DSA either as a standalone intervention or as an adjunct to conventional rehabilitation in adults with stroke. The primary outcome will be motor function. Secondary outcomes will include activities of daily living and adverse events, while exploratory outcomes will include neurological impairment, balance, spasticity, health-related quality of life, and depression. Two reviewers will independently conduct study selection, data extraction, and risk-of-bias assessment using the Cochrane Risk of Bias 2 tool. Where appropriate, meta-analyses will be performed using a random-effects model, and the certainty of evidence will be assessed using the Grading of Recommendations. Assessment, Development, and Evaluation approach. This systematic review is expected to provide up-to-date evidence on the efficacy and safety of DSA for stroke rehabilitation. The findings may inform clinical practice and policy, and help guide future research.

**Systematic review registration:**

https://www.crd.york.ac.uk/PROSPERO/view/CRD420251117520, Identifier CRD420251117520.

## 1 Introduction

Stroke is a leading cause of long-term disability worldwide and continues to impose a substantial burden on patients, caregivers, and healthcare systems (1, 2). Many stroke survivors experience persistent impairments in activities of daily living (ADL), mobility, cognition, and quality of life, often requiring prolonged rehabilitation and support (1, 2). Consequently, improving functional recovery after stroke remains a major clinical and public health priority.

Post-stroke rehabilitation is typically multidisciplinary, incorporating physiotherapy, occupational therapy, and other supportive interventions. Current evidence-based guidelines recommend coordinated rehabilitation that is initiated early after stroke and focused on intensive, task-specific training (3). Despite these recommendations, recovery outcomes remain highly variable, and many patients fail to regain their premorbid level of function (4). Moreover, no single rehabilitation strategy has demonstrated consistent superiority across all functional domains, and heterogeneity in study design and outcome assessment continues to hinder interpretation of the evidence.

Acupuncture has been investigated as a complementary intervention in stroke rehabilitation, with scalp acupuncture specifically designed to target cortical regions associated with motor and sensory function (5). Dynamic scalp acupuncture (DSA), also known as interactive DSA, is a task-integrated form of scalp acupuncture in which needling is combined with concurrent active movement or functional task performance (6–8). This approach is hypothesized to enhance activity-dependent neuroplasticity during treatment. Preliminary randomized trials have reported potential improvements in motor and cognitive outcomes, while neuroimaging studies have demonstrated associations with altered functional connectivity in motor-related networks (6–8). The mechanistic rationale for DSA is grounded in the principles of experience-dependent neuroplasticity following stroke, whereby repetitive, task-specific motor practice promotes reorganization of residual motor networks and facilitates functional recovery (9, 10). By combining scalp acupuncture –which has been associated with the restoration of normal motor patterns (11)—with concurrent active movement, DSA may more effectively engage sensorimotor integration and motor learning processes than passive needling alone. Repeated practice of functionally relevant tasks during treatment may provide an optimal environment for activity-dependent cortical reorganization and targeted remodeling of motor-related networks (12). However, interpretation of the existing literature is limited by substantial variability in intervention protocols, comparator groups, and methodological quality (5). Although previous reviews have examined scalp acupuncture for stroke rehabilitation, many have pooled diverse scalp acupuncture modalities rather than consistently evaluating DSA as a task-integrated subgroup within scalp acupuncture (5). A focused synthesis of the evidence may therefore help clarify the role of DSA in post-stroke rehabilitation, both as a standalone therapy and as an adjunct to conventional rehabilitation.

Accordingly, we will conduct a systematic review and meta-analysis of randomized controlled trials (RCTs) to evaluate the efficacy and safety of DSA for post-stroke rehabilitation.

## 2 Methods

### 2.1 Study registration

This systematic review protocol was developed in accordance with the Preferred Reporting Items for Systematic Reviews and Meta-Analysis Protocols (PRISMA-P) statement (13). The protocol was prospectively registered with the International Prospective Register of Systematic Reviews (PROSPERO) under the registration No. CRD420251117520.

### 2.2 Eligibility criteria

#### 2.2.1 Type of study and participants

This review will include only RCTs. Quasi- RCTs, crossover studies, and trials without a concurrent control group will be excluded. RCTs will be eligible regardless of the language of publication.

RCTs involving adult patients (≥18 years) diagnosed with ischemic or hemorrhagic stroke at any stage (acute, subacute, or chronic) will be included. Only full-text publications will be considered. Trials involving mixed neurological populations (e.g., stroke and non-stroke neurological disorders) will be eligible only if stroke-specific data can be extracted separately.

#### 2.2.2 Type of intervention

The intervention of interest will be DSA. For this review, DSA is operationally defined as a task-integrated scalp acupuncture intervention in which needle retention at scalp acupoints is accompanied by concurrent, active, and intentional patient participation in motor or functional task practice. Thus, the defining characteristic of DSA is the integration of scalp acupuncture with active task performance during needle retention, distinguishing it from conventional scalp acupuncture, in which patients typically rest passively (6). To be classified as DSA, an intervention must meet all of the following criteria:

- Needle retention at one or more recognized scalp acupoints. Any scalp acupoint will be considered acceptable, regardless of the specific treatment region.
- Concurrent, voluntary, and goal-directed patient engagement in active task performance during needle retention.
- A dynamic training component delivered for at least 30 min per session.
- A minimum treatment dose of at least 10 sessions administered over a period of at least 1 week.
- If electroacupuncture is used, it must be combined with task-specific training that satisfies all of the above criteria.

The minimum DSA dosing threshold was derived from published RCTs evaluating DSA (7, 8, 14, 15). Eligible concurrent tasks included active rehabilitation exercises, task-oriented functional training, and other therapist-guided or self-initiated activities requiring intentional patient participation. Interventions involving assisted or partially passive movement will also be considered eligible if they are performed in conjunction with active movements or are explicitly designed to facilitate progression to active movement. In contrast, interventions consisting exclusively of passive stretching or passive movement without active patient participation will be excluded.

Only studies that clearly satisfy all prespecified DSA criteria will be included. Studies meeting only some of these criteria will be excluded.

Studies will be eligible if they evaluate DSA either as a standalone intervention or as an adjunct to conventional rehabilitation, provided that the independent effect of DSA or its incremental effect beyond conventional rehabilitation could be evaluated. Trials using noninvasive stimulation modalities (e.g., laser acupuncture or acupressure) as a proxy for DSA will be excluded.

#### 2.2.3 Type of controls

Eligible comparisons are prespecified based on whether DSA is evaluated as a standalone intervention or as an adjunct to conventional rehabilitation. The following comparisons will be included: (1) DSA plus conventional rehabilitation versus the same conventional rehabilitation alone; (2) DSA alone versus sham DSA; (3) DSA plus conventional rehabilitation versus the same conventional rehabilitation plus sham DSA; and (4) DSA alone versus no-treatment control.

For this review, sham DSA is defined as a control intervention designed to mimic both core components of DSA—scalp acupuncture and concurrent active task performance—while withholding their presumed specific therapeutic effects. Accordingly, a comparator is considered a sham DSA only when both the acupuncture and the exercise/task components are sham or non-therapeutic (16). Sham scalp acupuncture may include non-acupoint needling, superficial or non-penetrating procedures, or inactive electroacupuncture devices. Similarly, sham exercise or task components may include very low-intensity exercise, movements directed to non-target body regions, or simple joint mobilizations lacking meaningful task specificity.

To minimize co-intervention bias, particularly in studies involving combined multimodal rehabilitation programs, all non-DSA co-interventions must be identical across study arms in type, intensity, and duration. This ensures that the only systematic difference between groups is the application of DSA. Studies featuring asymmetrical co-interventions or inseparable multimodal treatment packages without an appropriate comparator will be excluded from quantitative synthesis and reserved strictly for narrative synthesis. The modality of background rehabilitation will be evaluated as a prespecified subgroup analysis.

### 2.3 Type of outcome measure

Consistent with established stroke rehabilitation guidelines and consensus recommendations, multiple outcome domains will be assessed to evaluate intervention effectiveness (17, 18). To reduce multiplicity and enhance interpretability, outcomes will be prespecified and categorized as primary, secondary, and exploratory outcomes.

Additionally, outcome assessments will be categorized into short-term (<3 months post-randomization), medium-term (3–6 months post-randomization), and long-term (>6 months post-randomization). For studies reporting outcomes at multiple time points, the assessment closest to the end of the intervention period will be prioritized.

#### 2.3.1 Primary outcomes

The primary outcome will be motor function, assessed using validated instruments including the Fugl-Meyer Assessment, Motor Assessment Scale, and Action Research Arm Test.

#### 2.3.2 Secondary outcomes

Secondary outcomes include:

1. ADL, measured using the Modified Barthel Index, the Barthel Index, and the motor subscale of the Functional Independence Measure.
2. Incidence of all reported adverse events.

For safety outcomes, data will be extracted on the number and type of adverse events, serious adverse events, and treatment discontinuations due to adverse events, as reported in the included studies.

#### 2.3.3 Exploratory outcomes

Exploratory outcomes include:

1. Neurological impairment, assessed using the National Institutes of Health Stroke Scale.
2. Balance, assessed using the Berg Balance Scale and the Timed Up and Go test.
3. Spasticity, assessed using the Modified Ashworth Scale.
4. Health-related quality of life, assessed using the Stroke-Specific Quality of Life scale.
5. Depression, assessed using the Hamilton Depression Rating Scale and the Beck Depression Inventory.

### 2.4 Search methods for identification of studies

#### 2.4.1 Electronic data sources

A systematic literature search will be conducted in the following electronic databases: PubMed, Embase, Cochrane Central Register of Controlled Trials, Cumulative Index to Nursing and Allied Health Literature, China National Knowledge Infrastructure, Wanfang Data, Research Information Sharing Service, Korean Studies Information Service System, and Korean Medical Database. Each database will be searched from inception to October 20, 2025.

#### 2.4.2 Searching other resources

Reference lists of included studies and relevant systematic reviews will be manually screened to identify additional eligible trials. Grey literature will be retrieved from trial registries (ClinicalTrials.gov and the World Health Organization International Clinical Trials Registry Platform), conference proceedings of the World Federation of Acupuncture-Moxibustion Societies and the World Federation of Chinese Medicine Societies, and institutional repositories of relevant academic centers.

#### 2.4.3 Search strategy

The search strategy will combine Medical Subject Headings terms and free-text keywords related to “stroke” and DSA. Strategies will be adapted for each database. Because validated RCT search filters are not available for all databases, study design eligibility will be verified directly by the reviewers during screening. To minimize the risk of omitting eligible non-English records during the search process, the search terms for the respective databases will include both English keywords and their machine-translated equivalents in relevant target language. Full reproducible search strategies for all databases are provided in Supplementary File 1.

### 2.5 Data collection and analysis

#### 2.5.1 Selection of studies

Two reviewers (SA and WS) will independently screen all identified titles and abstracts. The full texts of potentially eligible studies will then be retrieved for detailed assessment. Any disagreements will be resolved through discussion or, if necessary, consultation with a third reviewer (HK). For other non-English records, where native proficiency cannot be guaranteed, we will utilize established machine translation tools for initial screening. In the absence of support from a native speaker or professional translator, records with uncertain eligibility will be retained for full-text assessment and evaluated conservatively by the review team. All non-English full-text articles will undergo the same dual independent screening and data extraction process applied to English-language studies.

**Figure 1:**
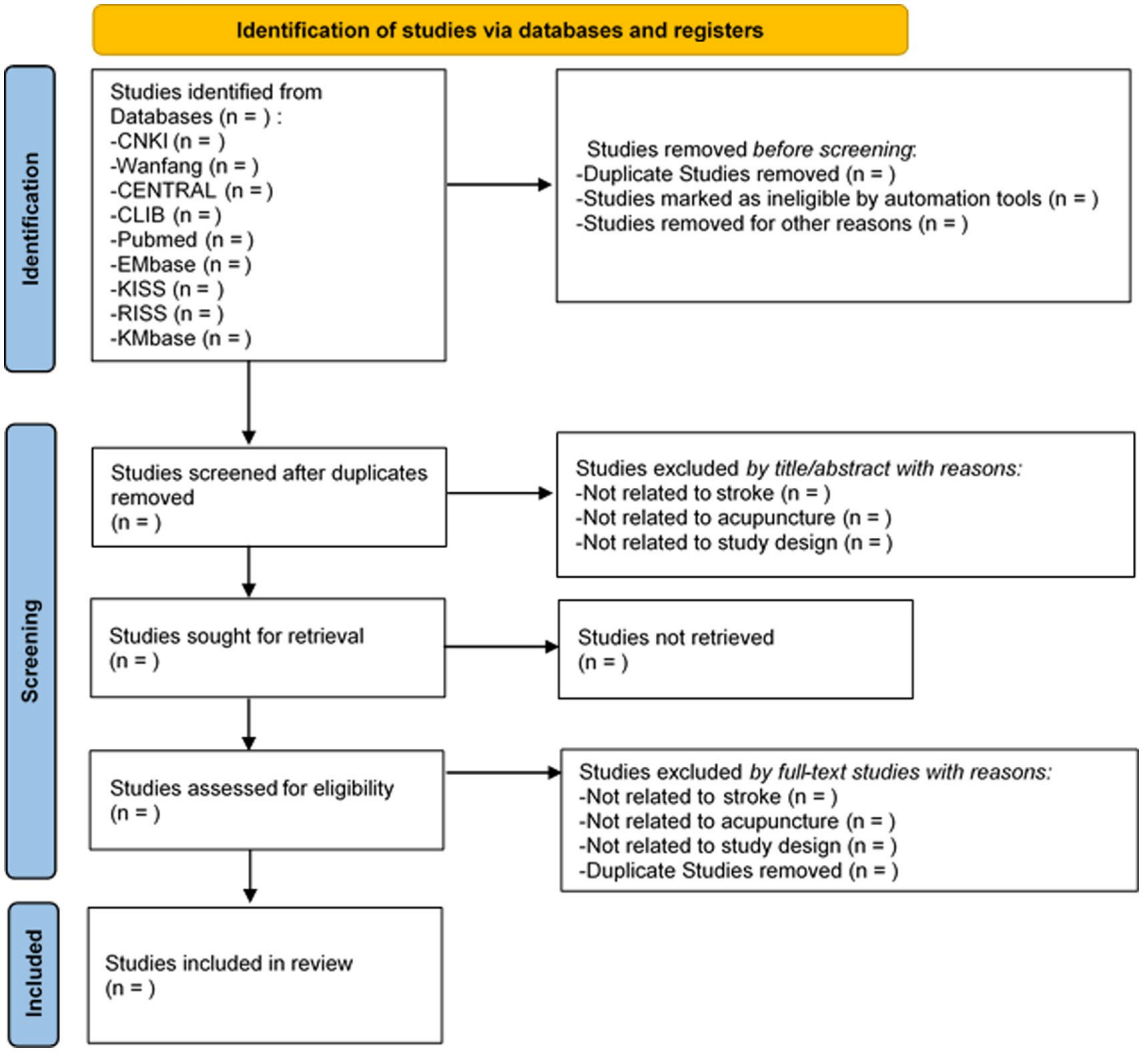
PRISMA 2020 flow diagram template for systematic review.

The study selection process is illustrated in the PRISMA flow diagram template (Figure 1).

#### 2.5.2 Data extraction and management

A standardized data extraction form will be developed and piloted before use. Two reviewers (SA and WS) will independently extract data, including study identifiers, participant characteristics, sample sizes, intervention and control details, outcomes, results, and risk-of-bias information. To ensure comprehensive reporting, details of the DSA protocol will be extracted according to the Standards for Reporting Interventions in Clinical Trials of Acupuncture (STRICTA) checklist (19). STRICTA will be used descriptively as a structured framework to enhance intervention characterization and reporting transparency; no quantitative reporting-quality scores will be calculated. When essential information is missing or unclear, attempts will be made to contact the corresponding authors for clarification. All records will be managed using EndNote 21 (Clarivate Analytics) for deduplication and citation tracking throughout the screening process.

Before formal screening, a calibration exercise will be conducted to ensure consistency between the two primary reviewers (SA and WS). Both reviewers will independently screen a random sample of 10% of the retrieved citations. Discrepancies will be discussed and resolved, with input from the third reviewer (HK) when necessary, before proceeding to full-text screening. In addition, the data extraction form will be piloted on three included studies to ensure consistency and refine the extraction process before formal data extraction begins.

#### 2.5.3 Assessment of risk of bias in included studies

The methodological quality of included RCTs will be assessed independently by two reviewers (SA and WS) using the revised Cochrane Risk of Bias 2 (RoB 2) tool (20). This tool evaluates five domains: bias arising from the randomization process, deviations from intended interventions, missing outcome data, outcome measurement, and selection of the reported result. Any disagreements will be resolved through discussion or consultation with a third reviewer (HK). The risk-of-bias assessments will be used to contextualize the review findings and inform sensitivity analyses evaluating the robustness of the review conclusions.

#### 2.5.4 Data synthesis

Continuous data will be pooled using mean differences when studies employ the same measurement scale and standardized mean differences when different scales are used. Dichotomous data will be pooled using odds ratios (Ors). All pooled effect estimates will be reported as 95% confidence intervals (CIs).

When multiple validated motor function instruments are reported within a single study, only one measure will be included in each meta-analysis to avoid selective outcome reporting. Outcome measure will be selected according to a predefined hierarchy: (1) the Fugl-Meyer Assessment, (2) the Motor Assessment Scale, and (3) the Action Research Arm Test.

As prespecified in the type of controls, outcomes will be synthesized separately within each comparison category. Pooled estimates from different comparison categories will not be combined into a single overall effect estimate. For multi-arm trials, shared control groups will be divided proportionally, or intervention arms will be combined to create a single pairwise comparison, with variance adjustments performed in accordance with Cochrane guidance (21).

Meta-analyses will be conducted using a random-effects model, given the anticipated clinical and methodological heterogeneity of the acupuncture interventions and study populations. To obtain more robust interval estimates, between-study variance (τ^2^) will be estimated using the Paule–Mandel estimator, and pooled-effect CIs will be calculated using the Hartung–Knapp–Sidik–Jonkman method for all primary meta-analyses. This approach provides improved control of Type I error, particularly when the number of included studies is small, while yielding results that are broadly comparable to conventional random-effects methos as the number of studies increases (22). Where meta-analysis is not feasible because of insufficient data or substantial methodological incompatibility, findings will be synthesized narratively.

Safety outcomes will be evaluated based on reported adverse events. Given the anticipated variability in adverse-event reporting, safety data will primarily be summarized descriptively. Meta-analysis of safety outcomes will be undertaken only when sufficient data are available. For studies containing a zero event count in one treatment arm, a continuity correction of 0.5 will be applied. Studies with zero events in both arms will be excluded from pooled analyses but retained in the descriptive synthesis.

All statistical analyses will be conducted using R (R Foundation for Statistical Computing, Vienna, Austria) with the “meta” package, alongside Review Manger (RevMan) version 5.4.1 (Cochrane Collaboration, Oxford, United Kingdom).

#### 2.5.5 Dealing with missing data

If essential data remain unavailable after attempts to contact the corresponding authors, meta-analyses will be restricted to outcomes with complete data, while studies lacking the necessary statistics will be included in the narrative synthesis. Only available data will be analyzed, and the potential impact of missing data on effect estimates and evidence certainty will be explicitly discussed. No statistical imputation of missing summary statistics will be performed.

#### 2.5.6 Assessment of heterogeneity

Between-study heterogeneity will be assessed using the I^2^ statistic and supplemented by visual inspection of forest plots. Meta-regression will be conducted only when substantial heterogeneity is detected (I^2^ > 50%) (23) and at least three studies are available for the outcome of interest. Analyses will be limited to prespecified candidate moderators identified *a priori* in the subgroup analysis plan, including stroke phase, stroke type, treatment duration, type of adjunctive rehabilitation or task component, and assessment timeframe. All meta-regression analyses will be explicitly designated as exploratory and reported using *β* coefficients, *R*^2^ values, and 95% CIs.

#### 2.5.7 Assessment of publication biases

If a meta-analysis includes at least 10 studies for a given outcome, publication bias will be assessed through visual inspection of funnel plots and formally evaluated using Egger’s regression test for continuous outcomes and Harbord’s test for dichotomous outcomes. Funnel plot asymmetry will be interpreted cautiously, as it may reflect heterogeneity, selective outcome reporting, chance, or other factor in addition to publication bias.

#### 2.5.8 Subgroup analysis

All planned subgroup analyses will be explicitly designated as exploratory and hypothesis-generating rather than confirmatory. These analyses will be conducted only when sufficient clinically meaningful data (i.e., at least two studies per subgroup) are available. To explore potential sources of heterogeneity, the following variables were prespecified *a priori* as the most plausible moderators of treatment effects, in order of priority: (1) stroke phase (acute, subacute, or chronic); (2) stroke type (ischemic or hemorrhagic); (3) treatment duration (≤4 weeks or >4 weeks); (4) type of adjunctive rehabilitation/task component (rehabilitation exercises, cognitive tasks, or functional training); and (5) assessment timeframe (short-term, medium-term, or long-term). Given the exploratory nature of these analyses, no formal statistical adjustments for multiplicity will be applied. Instead, subgroup findings will be interpreted with extreme caution, explicitly acknowledging the increased risk of Type I error associated with multiple testing. Any planned subgroup analyses lacking sufficient data will be omitted. These variables will also be considered as candidate moderators in meta-regression analyses when substantial heterogeneity is present; however, subgroup analyses and meta-regression serve complementary purposes and will therefore be reported and interpreted separately as exploratory analyses.

#### 2.5.9 Sensitivity analysis

Sensitivity analyses will be conducted to evaluate the robustness of the primary findings. Studies judged to have high overall risk of bias will be excluded, and the resulting estimates will be compared with those from the primary analyses. The DerSimonian–Laird estimator will also be applied to assess the sensitivity of results to the choice of between-study variance estimator. Where sufficient data are available, analyses will also be restricted to studies rated as low risk of bias or having some concerns in the outcome measurement domain of RoB 2 to evaluate the potential influence of detection bias.

All sensitivity analyses will be considered exploratory. Any substantial divergence from the primary estimates will be taken into account when determining the Grading of Recommendations, Assessment, Development, and Evaluation (GRADE) certainty ratings.

#### 2.5.10 Certainty of evidence

The overall certainty of evidence for each outcome will be assessed independently by two reviewers (SA and WS) using the GRADE approach (24). The certainty of evidence will be rated as high, moderate, low, or very low. Any disagreements will be resolved through consultation with a third reviewer (HK). A GRADE Summary of Findings table will be prepared to present the results (25).

#### 2.5.11 Patient and public involvement

Patients and members of the public were not involved in the design, conduct, reporting, or dissemination plans of this study.

## 3 Discussion

This protocol outlines a rigorous plan to synthesize evidence from RCTs evaluating the efficacy and safety of DSA in post-stroke rehabilitation. Given the persistent functional burden after stroke and the limitations of conventional rehabilitation alone, a comprehensive evaluation of DSA as either an adjunctive or standalone intervention, compared with sham or no treatment, is clinically relevant (1, 3, 4). DSA combines scalp acupuncture with concurrent task engagement to leverage activity-dependent neuroplasticity. Early multicenter trials and neuroimaging studies have reported potential benefits for ADL, cognition, mood, and functional connectivity; however, interpretation of these findings has remained limited by variability in intervention protocols and study quality (6, 7).

Previous meta-analyses of acute ischemic and hemorrhagic stroke cohorts generally pooled heterogeneous scalp acupuncture interventions and reported short-term benefits. However, their conclusions were constrained by small sample sizes, substantial heterogeneity, and inconsistent outcome reporting (26, 27). Subsequent syntheses addressing hemiparesis, spasticity, and post-stroke cognition also suggested favorable effects of scalp acupuncture relative to other acupuncture modalities, but the evidence remained limited by heterogeneous intervention protocols, diverse comparators, modest sample sizes, and short follow-up periods (28–30). Although some systematic reviews have included subgroup analyses of scalp acupuncture (5, 31), DSA has not been consistently evaluated as a distinct task-integrated intervention within the broader category of scalp acupuncture. Building on previous evidence syntheses, this review is intended as an incremental refinement of the existing evidence base by applying a clearer operational definition of DSA and, where feasible, evaluating it separately from non-dynamic scalp acupuncture. By restricting inclusion to RCTs with concurrent comparator groups and prespecified comparator categories, the review aims to more clearly characterize DSA-related effects while accounting for non-specific and background treatment effects. Methodological strengths include adherence to PRISMA-P guidelines, prospective registration in PROSPERO, application of the RoB 2 tool and GRADE frameworks, and comprehensive multilingual searches across major international and Asian databases. The review will also evaluate outcomes that are clinically relevant to post-stroke populations, including ADL, quality of life, emotional symptoms, and safety, thereby facilitating interpretation in clinical practice. If DSA demonstrates consistent benefits across prioritized outcome domains, particularly when used as an adjunct to conventional rehabilitation or when compared with sham or no treatment, the findings may help refine the evidence base for integrative rehabilitation strategies and identify priorities for future research. However, the review is not intended to establish a fundamentally novel conceptual framework or therapeutic paradigm. Accordingly, the findings should be considered exploratory and hypothesis-generating, and their interpretation should remain cautious.

Several limitations are anticipated. First, substantial clinical and methodological heterogeneity is expected across stroke phase and subtypes, baseline severity, co-interventions, and DSA delivery characteristics—including treatment location, modality, dose, and intensity—as well as outcome measures and follow-up durations. Although random-effects meta-analysis, prespecified subgroup or moderator analyses, and narrative synthesis will be applied where appropriate, the absence of standardized DSA protocols may limit direct comparability across studies and hinder interpretation of dose–response relationships. Second, blinding remains challenging in acupuncture research, increasing the risk of performance and detection bias. Standardizing sham DSA is particularly difficult because procedures intended as sham interventions, such as superficial needling or low-intensity exercise, may not be biologically inert and could still exert mild physiological or neuroplastic effects. Such “active shams” may reduce the observed differences between intervention and control groups, potentially leading to underestimation of the true treatment effect. To address this issue, comparator categories and sham-based comparisons will be analyzed separately where feasible, and sensitivity analyses will be restricted to assessor-blinded or otherwise lower-risk studies. Third, restricting the reviews to RCTs may limit the identification of rare or long-term adverse events. Although safety data will be extracted comprehensively, conclusions regarding infrequent harms and the durability of treatment effects should be interpreted cautiously. Fourth, small sample sizes, short follow-up periods, and geographic clustering of studies may contribute to imprecision and increase the risk of small-study effects and location bias. To mitigate these concerns, broad multilingual searches and assessments of small-study effects will be conducted where feasible. Finally, incomplete adherence to STRICTA and inconsistent reporting and variable descriptions of the dynamic task component may limit assessment of intervention fidelity and mechanistic interpretation. Extraction of STRICTA-concordant reporting items will improve transparency, and any residual uncertainty will be reflected in the GRADE assessments. Consequently, any conclusions drawn from this review will be viewed as refinements of the existing scalp acupuncture rehabilitation literature rather than evidence supporting a new rehabilitation paradigm.
