# Supplementary material for: Assessing efficacy and safety of dynamic scalp acupuncture for post-stroke rehabilitation: systematic review and meta-analysis protocol
Source: Front Neurol. 2026 Jul 17;17:1750422. doi: 10.3389/fneur.2026.1750422 (PMC13423972; doi:10.3389/fneur.2026.1750422)
Supplement: Supplementary file 1 [file Table_1.DOCX]

Supplementary file 1. Search Strategy Used in Each Database.

MEDLINE via PubMed

| #1 | Stroke[MeSH Terms] |
| --- | --- |
| #2 | ("Stroke" OR "Cerebrovascular Accident" OR "Brain Infarction" OR "Cerebral Infarction" OR "Brain Stem Infarction*" OR "Hemorrhagic Stroke" OR "Ischemic Stroke" OR "Embolic Stroke" OR "Thrombotic Stroke" OR "Lacunar Stroke" OR "Multi-Infarct Dementia" OR "Anterior Cerebral Artery Infarction" OR "Middle Cerebral Artery Infarction" OR "Posterior Cerebral Artery Infarction" OR "Lateral Medullary Syndrome" OR CADASIL)[Title/Abstract] |
| #3 | #1 OR #2 |
| #4 | (Acupuncture[Mesh]) OR (Acupuncture Therapy[Mesh]) OR (Dry needling[Mesh]) OR (Meridians[Mesh]) |
| #5 | (Acupuncture[Title/Abstract]) OR (Acupuncture Therapy[Title/Abstract]) OR (Dry needling[Title/Abstract]) OR (Meridians[Title/Abstract]) OR (Dynamic Scalp Acupuncture[Title/Abstract]) OR (Interactive Dynamic Scalp Acupuncture[Title/Abstract]) |
| #6 | #4 OR #5 |
| #7 | #3 AND #6 |

Embase via Elsevier.com

| #1 | 'cerebrovascular accident'/exp |
| --- | --- |
| #2 | (('brain' OR 'cerebrovascular' OR 'cerebral') NEXT/1 (infarct* OR ischemi* OR hemorrha* OR accident)) OR 'stroke':ti,ab,kw |
| #3 | #1 OR #2 |
| #4 | 'acupuncture'/exp OR 'dry needling'/exp OR 'body meridian'/exp OR 'trigger point'/exp |
| #5 | 'acupuncture':ab,ti,kw OR 'dry needling':ab,ti,kw OR 'body meridian':ab,ti,kw OR 'trigger point':ab,ti,kw OR 'dynamic scalp acupuncture':ab,ti,kw OR ‘interactive dynamic scalp acupuncture’:ab,ti,kw |
| #6 | #4 OR #5 |
| #7 | #3 AND #6 |

CINAHL

| #1 | MH "Stroke+" |
| --- | --- |
| #2 | XB ("Stroke" OR "Cerebrovascular Accident" OR "Brain Infarction" OR "Cerebral Infarction" OR "Brain Stem Infarction*" OR "Hemorrhagic Stroke" OR "Ischemic Stroke" OR "Embolic Stroke" OR "Thrombotic Stroke" OR "Lacunar Stroke" OR "Multi-Infarct Dementia" OR "Anterior Cerebral Artery Infarction" OR "Middle Cerebral Artery Infarction" OR "Posterior Cerebral Artery Infarction" OR "Lateral Medullary Syndrome" OR CADASIL) |
| #3 | #1 OR #2 |
| #4 | MH "Acupuncture+" OR MH "Meridians+" |
| #5 | XB ( Acupuncture OR "Acupuncture Therapy" OR "Dry Needling" OR "Trigger Point" OR Meridians OR "Dynamic Scalp Acupuncture" OR "Interactive Dynamic Scalp Acupuncture" ) |
| #6 | #4 OR #5 |
| #7 | #3 AND #6 |

CENTRAL

| #1 | MeSH descriptor: [Stroke] Explode all trees |
| --- | --- |
| #2 | ("Stroke" OR "Cerebrovascular Accident" OR "Brain Infarction" OR "Cerebral Infarction" OR "Brain Stem Infarction*" OR "Hemorrhagic Stroke" OR "Ischemic Stroke" OR "Embolic Stroke" OR "Thrombotic Stroke" OR "Lacunar Stroke" OR "Multi-Infarct Dementia" OR "Anterior Cerebral Artery Infarction" OR "Middle Cerebral Artery Infarction" OR "Posterior Cerebral Artery Infarction" OR "Lateral Medullary Syndrome" OR CADASIL):ti,ab,kw |
| #3 | #1 OR #2 |
| #4 | MeSH descriptor: [Dry Needling] Explode all trees |
| #5 | MeSH descriptor: [Acupuncture Therapy] Explode all trees |
| #6 | MeSH descriptor: [Meridians] Explode all trees |
| #7 | #4 OR #5 OR #6 |
| #8 | ('Acupuncture Therapy' OR 'Dry Needling' OR ‘Meridians’ OR ‘Dynamic Scalp Acupuncture’ OR ‘Interactive Dynamic Scalp Acupuncture’):ti,ab,kw |
| #9 | #7 OR #8 |
| #10 | #3 AND #9 |

CNKI professional search

| #1 | TI='Stroke' OR TI='Cerebrovascular Accident' OR TI='Brain Infarction' OR TI='Cerebral Infarction' OR TI='Brain Stem Infarction*' OR TI='Hemorrhagic Stroke' OR TI='Ischemic Stroke' OR TI='Embolic Stroke' OR TI='Thrombotic Stroke' OR TI='Lacunar Stroke' OR TI='Multi-Infarct Dementia' OR TI='Anterior Cerebral Artery Infarction' OR TI='Middle Cerebral Artery Infarction' OR TI='Posterior Cerebral Artery Infarction' OR TI='Lateral Medullary Syndrome' OR TI='CADASIL' |
| --- | --- |
| #2 | AB='Stroke' OR AB='Cerebrovascular Accident' OR AB='Brain Infarction' OR AB='Cerebral Infarction' OR AB='Brain Stem Infarction*' OR AB='Hemorrhagic Stroke' OR AB='Ischemic Stroke' OR AB='Embolic Stroke' OR AB='Thrombotic Stroke' OR AB='Lacunar Stroke' OR AB='Multi-Infarct Dementia' OR AB='Anterior Cerebral Artery Infarction' OR AB='Middle Cerebral Artery Infarction' OR AB='Posterior Cerebral Artery Infarction' OR AB='Lateral Medullary Syndrome' OR AB='CADASIL' |
| #3 | KY='Stroke' OR KY='Cerebrovascular Accident' OR KY='Brain Infarction' OR KY='Cerebral Infarction' OR KY='Brain Stem Infarction*' OR KY='Hemorrhagic Stroke' OR KY='Ischemic Stroke' OR KY='Embolic Stroke' OR KY='Thrombotic Stroke' OR KY='Lacunar Stroke' OR KY='Multi-Infarct Dementia' OR KY='Anterior Cerebral Artery Infarction' OR KY='Middle Cerebral Artery Infarction' OR KY='Posterior Cerebral Artery Infarction' OR KY='Lateral Medullary Syndrome' OR KY='CADASIL' |
| #4 | #1 OR #2 OR #3 |
| #5 | TI='acupuncture' OR TI='acupuncture therapy' OR TI='dry needling' OR TI=‘meridians’ OR TI='dynamic scalp acupuncture' OR TI=’interactive dynamic scalp acupuncture’ |
| #6 | AB='acupuncture' OR AB='acupuncture therapy' OR AB='dry needling' OR AB=‘meridians’ OR AB='dynamic scalp acupuncture' OR AB=’interactive dynamic scalp acupuncture’ |
| #7 | KY='acupuncture' OR KY='acupuncture therapy' OR KY='dry needling' OR KY=‘meridians’ OR KY='dynamic scalp acupuncture' OR KY=’interactive dynamic scalp acupuncture’ |
| #8 | #5 OR #6 OR #7 |
| #9 | #4 AND #8 |
| #10 | TI='中风' OR TI='脑卒中' OR TI='卒中' OR TI='脑血管意外' OR TI='脑梗死' OR TI='脑梗塞' OR TI='脑干梗死' OR TI='出血性脑卒中' OR TI='脑出血' OR TI='缺血性脑卒中' OR TI='脑栓塞' OR TI='脑血栓形成' OR TI='腔隙性脑梗死' OR TI='多发梗死性痴呆' OR TI='大脑前动脉梗死' OR TI='大脑中动脉梗死' OR TI='大脑后动脉梗死' OR TI='延髓背外侧综合征' OR TI='CADASIL' |
| #11 | AB='中风' OR AB='脑卒中' OR AB='卒中' OR AB='脑血管意外' OR AB='脑梗死' OR AB='脑梗塞' OR AB='脑干梗死' OR AB='出血性脑卒中' OR AB='脑出血' OR AB='缺血性脑卒中' OR AB='脑栓塞' OR AB='脑血栓形成' OR AB='腔隙性脑梗死' OR AB='多发梗死性痴呆' OR AB='大脑前动脉梗死' OR AB='大脑中动脉梗死' OR AB='大脑后动脉梗死' OR AB='延髓背外侧综合征' OR AB='CADASIL' |
| #12 | KY='中风' OR KY='脑卒中' OR KY='卒中' OR KY='脑血管意外' OR KY='脑梗死' OR KY='脑梗塞' OR KY='脑干梗死' OR KY='出血性脑卒中' OR KY='脑出血' OR KY='缺血性脑卒中' OR KY='脑栓塞' OR KY='脑血栓形成' OR KY='腔隙性脑梗死' OR KY='多发梗死性痴呆' OR KY='大脑前动脉梗死' OR KY='大脑中动脉梗死' OR KY='大脑后动脉梗死' OR KY='延髓背外侧综合征' OR KY='CADASIL' |
| #13 | #10 OR #11 OR #12 |
| #14 | TI='针刺' OR TI='针灸' OR TI='针刺疗法' OR TI='针灸疗法' OR TI='干针' OR TI='经络' OR TI='头针' OR TI='头皮针' OR TI='动态头针' OR TI='动留针' OR TI='互动式头针' |
| #15 | AB='针刺' OR AB='针灸' OR AB='针刺疗法' OR AB='针灸疗法' OR AB='干针' OR AB='经络' OR AB='头针' OR AB='头皮针' OR AB='动态头针' OR AB='动留针' OR AB='互动式头针' |
| #16 | KY='针刺' OR KY='针灸' OR KY='针刺疗法' OR KY='针灸疗法' OR KY='干针' OR KY='经络' OR KY='头针' OR KY='头皮针' OR KY='动态头针' OR KY='动留针' OR KY='互动式头针' |
| #17 | #14 OR #15 OR #16 |
| #18 | #13 AND #17 |
| #19 | #9 OR #18 |

Wanfang

| #1 | Title=Stroke OR Title=Cerebrovascular Accident OR Title=Brain Infarction OR Title=Cerebral Infarction OR Title=Brain Stem Infarction* OR Title=Hemorrhagic Stroke OR Title=Ischemic Stroke OR Title=Embolic Stroke OR Title=Thrombotic Stroke OR Title=Lacunar Stroke OR Title=Multi-Infarct Dementia OR Title=Anterior Cerebral Artery Infarction OR Title=Middle Cerebral Artery Infarction OR Title=Posterior Cerebral Artery Infarction OR Title=Lateral Medullary Syndrome OR Title=CADASIL |
| --- | --- |
| #2 | Abstract=Stroke OR Abstract=Cerebrovascular Accident OR Abstract=Brain Infarction OR Abstract=Cerebral Infarction OR Abstract=Brain Stem Infarction* OR Abstract=Hemorrhagic Stroke OR Abstract=Ischemic Stroke OR Abstract=Embolic Stroke OR Abstract=Thrombotic Stroke OR Abstract=Lacunar Stroke OR Abstract=Multi-Infarct Dementia OR Abstract=Anterior Cerebral Artery Infarction OR Abstract=Middle Cerebral Artery Infarction OR Abstract=Posterior Cerebral Artery Infarction OR Abstract=Lateral Medullary Syndrome OR Abstract=CADASIL |
| #3 | Keyword=Stroke OR Keyword=Cerebrovascular Accident OR Keyword=Brain Infarction OR Keyword=Cerebral Infarction OR Keyword=Brain Stem Infarction* OR Keyword=Hemorrhagic Stroke OR Keyword=Ischemic Stroke OR Keyword=Embolic Stroke OR Keyword=Thrombotic Stroke OR Keyword=Lacunar Stroke OR Keyword=Multi-Infarct Dementia OR Keyword=Anterior Cerebral Artery Infarction OR Keyword=Middle Cerebral Artery Infarction OR Keyword=Posterior Cerebral Artery Infarction OR Keyword=Lateral Medullary Syndrome OR Keyword=CADASIL |
| #4 | #1 OR #2 OR #3 |
| #5 | Title=acupuncture OR Title=acupuncture therapy OR Title=dry needling OR Title=meridians OR Title=dynamic scalp acupuncture OR Title=interactive dynamic scalp acupuncture |
| #6 | Abstract=acupuncture OR Abstract=acupuncture therapy OR Abstract=dry needling OR Abstract=meridians OR Abstract=dynamic scalp acupuncture OR Abstract=interactive dynamic scalp acupuncture |
| #7 | Keyword=acupuncture OR Keyword=acupuncture therapy OR Keyword=dry needling OR Keyword=meridians OR Keyword=dynamic scalp acupuncture OR Keyword=interactive dynamic scalp acupuncture |
| #8 | #5 OR #6 OR #7 |
| #9 | #4 AND #8 |
| #10 | Title=中风 OR Title=脑卒中 OR Title=卒中 OR Title=脑血管意外 OR Title=脑梗死 OR Title=脑梗塞 OR Title=脑干梗死 OR Title=出血性脑卒中 OR Title=脑出血 OR Title=缺血性脑卒中 OR Title=脑栓塞 OR Title=脑血栓形成 OR Title=腔隙性脑梗死 OR Title=多发梗死性痴呆 OR Title=大脑前动脉梗死 OR Title=大脑中动脉梗死 OR Title=大脑后动脉梗死 OR Title=延髓背外侧综合征 OR Title=CADASIL |
| #11 | Abstract=中风 OR Abstract=脑卒中 OR Abstract=卒中 OR Abstract=脑血管意外 OR Abstract=脑梗死 OR Abstract=脑梗塞 OR Abstract=脑干梗死 OR Abstract=出血性脑卒中 OR Abstract=脑出血 OR Abstract=缺血性脑卒中 OR Abstract=脑栓塞 OR Abstract=脑血栓形成 OR Abstract=腔隙性脑梗死 OR Abstract=多发梗死性痴呆 OR Abstract=大脑前动脉梗死 OR Abstract=大脑中动脉梗死 OR Abstract=大脑后动脉梗死 OR Abstract=延髓背外侧综合征 OR Abstract=CADASIL |
| #12 | Keyword=中风 OR Keyword=脑卒中 OR Keyword=卒中 OR Keyword=脑血管意外 OR Keyword=脑梗死 OR Keyword=脑梗塞 OR Keyword=脑干梗死 OR Keyword=出血性脑卒中 OR Keyword=脑出血 OR Keyword=缺血性脑卒中 OR Keyword=脑栓塞 OR Keyword=脑血栓形成 OR Keyword=腔隙性脑梗死 OR Keyword=多发梗死性痴呆 OR Keyword=大脑前动脉梗死 OR Keyword=大脑中动脉梗死 OR Keyword=大脑后动脉梗死 OR Keyword=延髓背外侧综合征 OR Keyword=CADASIL |
| #13 | #10 OR #11 OR #12 |
| #14 | Title=针刺 OR Title=针灸 OR Title=针刺疗法 OR Title=针灸疗法 OR Title=干针 OR Title=经络 OR Title=头针 OR Title=头皮针 OR Title=动态头针 OR Title=动留针 OR Title=互动式头针 |
| #15 | Abstract=针刺 OR Abstract=针灸 OR Abstract=针刺疗法 OR Abstract=针灸疗法 OR Abstract=干针 OR Abstract=经络 OR Abstract=头针 OR Abstract=头皮针 OR Abstract=动态头针 OR Abstract=动留针 OR Abstract=互动式头针 |
| #16 | Keyword=针刺 OR Keyword=针灸 OR Keyword=针刺疗法 OR Keyword=针灸疗法 OR Keyword=干针 OR Keyword=经络 OR Keyword=头针 OR Keyword=头皮针 OR Keyword=动态头针 OR Keyword=动留针 OR Keyword=互动式头针 |
| #17 | #14 OR #15 OR #16 |
| #18 | #13 AND #17 |
| #19 | #9 OR #18 |

RISS

| #1 | ('Stroke' <OR> 'Cerebrovascular Accident' <OR> 'Brain Infarction' <OR> 'Cerebral Infarction' <OR> 'Brain Stem Infarction*' <OR> 'Hemorrhagic Stroke' <OR> 'Ischemic Stroke' <OR> 'Embolic Stroke' <OR> 'Thrombotic Stroke' <OR> 'Lacunar Stroke' <OR> 'Multi-Infarct Dementia' <OR> 'Anterior Cerebral Artery Infarction' <OR> 'Middle Cerebral Artery Infarction' <OR> 'Posterior Cerebral Artery Infarction' <OR> 'Lateral Medullary Syndrome' <OR> CADASIL) <AND> (Acupuncture <OR> 'Acupuncture Therapy' <OR> 'Dry Needling' <OR> 'Trigger Point' <OR> Meridians <OR> 'Dynamic Scalp Acupuncture' <OR> 'Interactive Dynamic Scalp Acupuncture') |
| --- | --- |
| #2 | (뇌졸중 <OR> 뇌경색 <OR> 뇌출혈 <OR> 일과성허혈발작 <OR> 뇌내출혈 <OR> 거미막밑출혈 <OR> 경막외출혈 <OR> 경막하출혈 <OR> 중풍) <AND> (침 <OR> ‘통증 유발점’ <OR> ‘침 치료’ <OR> 혈자리 <OR> 침술 <OR> 침법) |
| #3 | #1 OR #2 |

KISS

| #1 | ('Stroke' OR 'Cerebrovascular Accident' OR 'Brain Infarction' OR 'Cerebral Infarction' OR 'Brain Stem Infarction*' OR 'Hemorrhagic Stroke' OR 'Ischemic Stroke' OR 'Embolic Stroke' OR 'Thrombotic Stroke' OR 'Lacunar Stroke' OR 'Multi-Infarct Dementia' OR 'Anterior Cerebral Artery Infarction' OR 'Middle Cerebral Artery Infarction' OR 'Posterior Cerebral Artery Infarction' OR 'Lateral Medullary Syndrome' OR CADASIL) AND (Acupuncture OR 'Acupuncture Therapy' OR 'Dry Needling' OR 'Trigger Point' OR Meridians OR 'Dynamic Scalp Acupuncture' OR 'Interactive Dynamic Scalp Acupuncture') |
| --- | --- |
| #2 | (뇌졸중 OR 뇌경색 OR 뇌출혈 OR 일과성허혈발작 OR 뇌내출혈 OR 거미막밑출혈 OR 경막외출혈 OR 경막하출혈 OR 중풍) AND (침 OR ‘통증 유발점’ OR ‘침 치료’ OR 혈자리 OR 침술 OR 침법) |
| #3 | #1 OR #2 |

KMbase

| #1 | ([ALL=Stroke] OR [ALL=Cerebrovascular Accident] OR [ALL=Brain Infarction] OR [ALL=Cerebral Infarction] OR [ALL=Brain Stem Infarction*] OR [ALL=Hemorrhagic Stroke] OR [ALL=Ischemic Stroke] OR [ALL=Embolic Stroke] OR [ALL=Thrombotic Stroke] OR [ALL=Lacunar Stroke] OR [ALL=Multi-Infarct Dementia] OR [ALL=Anterior Cerebral Artery Infarction] OR [ALL=Middle Cerebral Artery Infarction] OR [ALL=Posterior Cerebral Artery Infarction] OR [ALL=Lateral Medullary Syndrome] OR [ALL=CADASIL]) AND ([ALL=Acupuncture] OR [ALL=Acupuncture Therapy] OR [ALL=Dry Needling] OR [ALL=Trigger Point] OR [ALL=Meridians] OR [ALL=Dynamic Scalp Acupuncture] OR [ALL=Interactive Dynamic Scalp Acupuncture]) |
| --- | --- |
| #2 | ([ALL=뇌졸중] OR [ALL=뇌경색] OR [ALL=뇌출혈] OR [ALL=일과성허혈발작] OR [ALL=뇌내출혈] OR [ALL=거미막밑출혈] OR [ALL=경막외출혈] OR [ALL=경막하출혈] OR [ALL=중풍]) AND ([ALL=침] OR [ALL=통증 유발점] OR [ALL=침 치료] OR [ALL=혈자리] OR [ALL=침술] OR [ALL=침법]) |
| #3 | #1 OR #2 |
